# Molecular Simulation of the Adsorption Characteristics of Methane in Pores of Coal with Different Metamorphic Degrees

**DOI:** 10.3390/molecules26237217

**Published:** 2021-11-28

**Authors:** Qing Han, Cunbao Deng, Zhixin Jin, Tao Gao

**Affiliations:** College of Safety and Emergency Management and Engineering, Taiyuan University of Technology, Jinzhong 030600, China; dengcunbao@tyut.edu.cn (C.D.); jinzhixin@tyut.edu.cn (Z.J.); gaotao0174@link.tyut.edu.cn (T.G.)

**Keywords:** coal molecular model, GCMC simulation, adsorption, different metamorphic degrees, enhanced gas recovery

## Abstract

In order to study differences in the methane adsorption characteristics of coal pores of different metamorphic degrees, 4 nm pore structure models based on three typical coal structure models with different metamorphic degrees were constructed. Based on the molecular mechanics and dynamics theory, the adsorption characteristics of methane in different coal rank pores were simulated by the grand canonical Monte Carlo (GCMC) and molecular dynamics methods. The isothermal adsorption curve, Van der Waals energy, concentration distribution, and diffusion coefficient of methane under different conditions were analyzed and calculated. The results showed that at the same pore size, the adsorption capacity of CH_4_ is positively correlated with pressure and metamorphic degree of coal, and the adsorption capacity of CH_4_ in high metamorphic coal is more affected by temperature. The relative concentration of CH_4_ in high-order coal pores is low, and the relative concentration at higher temperature and pressure conditions is high. The CH_4_ diffusion coefficient in high-rank coal is low, corresponding to the strong Van der Waals interaction between CH_4_ and coal. The research results are of great significance for further exploration of the interaction mechanism between CH_4_ and coal with different metamorphic degrees and can provide theoretical support for the selection of gas extraction parameters.

## 1. Introduction

China is rich in CBM resources, and many studies have shown that CBM reserves at a burial depth of less than 2000 m in China can reach 36.8 trillion m^3^ [1]. However, the coal reservoir permeability of most mining areas in China is low, resulting in a low gas extraction rate. At the same time, the risk of coal and gas outbursts in coal seams with low permeability is higher, which poses a severe threat to mine safety production. Coal is a complex material composed of inorganic matter and organic matter, which has complex internal surface structure characteristics and contains many pore structures. Because they are prominent sites of methane, it is of great significance to study the adsorption, desorption, seepage, and diffusion laws of pore structures in pore channels to improve gas extraction efficiency and prevent coal and gas outbursts. The occurrence states of methane in coal are adsorbed state and free state [2], and the adsorbed state methane is mainly adsorbed on the surface of coal and stored in coal pores. Therefore, taking measures to convert the adsorbed state methane into the free state and improve the permeability of coal itself is an effective method to improve the gas extraction rate [3].

The adsorption of methane in coal is dominated by physical adsorption, which is essentially the interaction between coal and methane molecules. The main adsorption site in this process is the pore structure of coal. In recent years, the accurate study of the adsorption mechanism of methane in coal pores by molecular simulation has been widely undertaken by scholars. However, the structure of the coal reservoir is complex, and the adsorption characteristics of methane are easily affected by many factors such as temperature [4], pressure [5], pore size [6,7], and coal metamorphic degree [8]. To further explore the interaction mechanism of coal and methane, different types of coal molecular structure models were constructed through a large number of research methods, among which the typical lignite structure models include the Wender [9], Shinn [10], and Hatcher models [11]. Bituminous coal models include the Wiser [12], Fuchs [13], Given [14], and Hill and Lyon models [15]. The anthracite models include the Hirsch [16], Pappano [17], and Xiang models [18].

On the molecular simulation of gas adsorption and desorption in the coal matrix, scholars at home and abroad have carried out systematic studies from many aspects. Gao et al. compared the difference between adsorption of a single component, binary, and ternary gas mixtures in lignite under different temperature conditions. The results showed that the isothermal adsorption heat of the adsorbate gas decreased with the increase in temperature [19]. Mosher et al. found that pressure had an essential influence on the competitive adsorption of CH_4_ and CO_2_ in the microporous and mesoporous structures of coal [20]. Liu et al. studied the effect of temperature on methane adsorption and gas adsorption density in pores with different pore sizes by molecular simulation [4]. Yin et al. constructed the pore structure model of coal with a specific diameter and shape by diamond filling and studied the motion state of methane in the pore structure and the influence of water content on the adsorption effect [21]. Tao et al. systematically simulated the adsorption and diffusion of CH_4_ and the intruding gases in coal via Monte Carlo and pointed out that the optimization injection depths of CO_2_ and N_2_ were 800–1100 and 600–900 m, respectively [22]. Asif et al. used MATLAB software to predict the competitive adsorption and diffusion characteristics of multi-component gases in coal with different coal ranks, showing that the competitive adsorption will be affected by the functional group structure of coal [23]. Bai et al. researched the desorption characteristics of CH_4_ after CO_2_ and N_2_ injection based on the configuration of coal-adsorbed methane and found that CO_2_ and N_2_ were mainly used to drive off methane by occupying adsorption sites [24]. Xiang et al. constructed coal macromolecular structure by molecular mechanics and molecular dynamics methods and explored the methane adsorption state in coal pore structure [18]. The competitive adsorption differences of different gas components injected into the goaf and the influence of moisture content on gas adsorption characteristics were explored by Wu et al. by using the GCMC method [25,26]. Yang et al. compared the differences in methane adsorption and desorption at different pore sizes and different water contents in coal reservoirs. They found that the superposition effect of the pore wall led to a large degree of difference in adsorption [27].

Most of the related studies above were based on the macromolecular model of coal to construct the coal matrix model with a specific pore structure, which does not fully consider the occurrence and flow state of methane in the pore channel. At the same time, most simulations of the adsorption process simplified the model and could not fully reflect the adsorption process. Therefore, it is necessary to construct a pore structure model that can reflect the actual situation of coal. Based on this conclusion, we selected three typical coal structural models with different metamorphic degrees at the present stage, including the Wender model, Wiser model, and Xiang model, which represented the structures of lignite, bituminous, and anthracite, respectively. At the same time, a 4nm pore structure was constructed as the methane migration channel, and the differences in the adsorption characteristics of pore methane in coal with different metamorphic degrees at different temperatures and pressures were analyzed to provide a theoretical basis for further exploration of the interaction mechanism between gas molecules and coal.

## 2. Coal Structure Model and Simulation Scheme

### 2.1. Establishment of Coal Model

**Figure 1:**
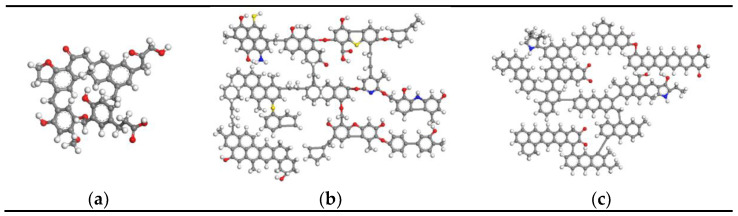
Basic structural units of coal: (**a**) Wender model, (**b**) Wiser model, (**c**) Xiang model.

Because of the complex structure of coal, it has no uniform configuration, and this study drew on the existing typical coal structure model to establish the corresponding coal pore structure model. The three typical coal structure models used in this study were the Wender lignite model, Wiser bituminous coal model, and Xiang anthracite model [18,28,29]. These models were constructed with Material Studio 8.0 software. The above models fully considered the three-dimensional structural characteristics of coal, and the percentage content of each element was in line with the actual situation. The basic structural units of the three coals are shown in Figure 1.

**Figure 2:**
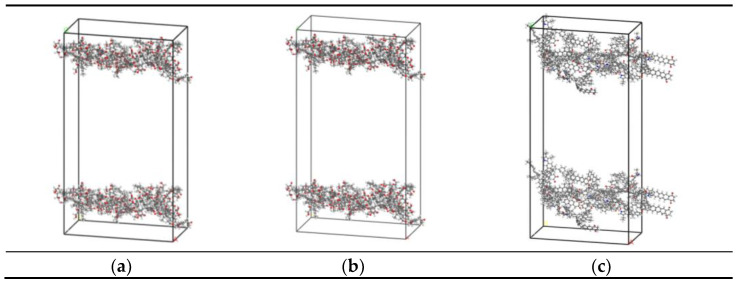
Coal pore (4 nm) structure models: (**a**) Wender model, (**b**) Wiser model, (**c**) Xiang model).

To truly reflect the pore structure of coal, 15 optimized lignite molecules, 10 bituminous coal molecules, and 5 anthracite molecules were placed into empty boxes. The periodic boundary conditions were added to construct the amorphous macromolecular structure model of coal. The completed model structures were optimized by geometric optimization and annealing kinetics to minimize the global energy. Then, the cell was expanded to a 2×1×1 supercell, and the 4nm vacuum layer was added to simulate the coal pore structure. The constructed coal pore structure model is shown in Figure 2. The densities corresponding to the lowest energy of the system were 1.19, 1.27, and 1.81 g/cm^3^ for bituminous, lignite, and anthracite, respectively. Additionally, the above values were close to the existing research results. Therefore, the optimized coal molecular spatial structure was reasonable.

### 2.2. Type of Force Field

The force field is the core of the simulation operation. The COMPASS force field is often used to simulate organic and inorganic molecules, which can accurately predict the properties of molecules and polymers. At the same time, many researchers have found that the COMPASS force field can be used to simulate the adsorption and diffusion properties of small gas molecules in macromolecules such as coal and shale. The calculation formula of system energy is shown in Formula (1) [30].

(1) $$E_{\mathrm{total}}=E_{\mathrm{valence}}+E_{\mathrm{crossterm}}+E_{\mathrm{non}-\mathrm{bond}}$$

In the formula, E_total_ represents the total energy of the system, E_valence_ represents the bonding energy, E_crossterm_ represents the covalent cross term, and E_non-bond_ represents the non-bond energy. The calculation method of E_valence_ is shown in Formula (2) [30].

(2) $$E_{\mathrm{valence}}=E_{\mathrm{bond}}+E_{\mathrm{angle}}+E_{\mathrm{torsion}}+E_{\mathrm{oop}}+E_{\mathrm{UB}}$$

In the formula, E_bond_ represents the bond stretching energy, E_angle_ represents the bond bending energy, E_torsion_ represents the corner torsion energy, E_oop_ represents the off-plane interaction energy, E_UB_ is the Urey–Bradley term.

The calculation method of non-bond energy E_non-bond_ is shown in Formula (3) [30].

(3) $$E_{\mathrm{non}-\mathrm{bon}}{}_{d}=E_{\mathrm{vdW}}+E_{\mathrm{ele}}+E_{\mathrm{hbond}}$$

In the formula, E_vdW_ represents Van der Waals interaction energy, E_ele_ represents electrostatic interaction energy, E_hbond_ represents hydrogen bond interaction energy.

### 2.3. Simulation Scheme

For methane adsorption in coal pores, the adsorption process was simulated by the GCMC method, which is mainly realized by adsorption isotherm and fixed pressure in the Sorption module. The adsorption pressure was between 1– and 5 MPa, and the temperature was set to 283.15, 303.15, and 333.15 K for bituminous, lignite, and anthracite, respectively. The equilibrium steps were 1×10^5^ steps, and the production steps were 1×10^6^ steps. The configuration calculation method was the metropolis method, the charge calculation method was forcefield assignment, the electrostatic calculation method was Ewald, and the accuracy was 1×10^−4^ kJ/mol. The Van der Waals interaction calculation method was atom-based, and the truncation radius was set to 1.55 nm. Since the sorption module calculates the adsorption amount by fugacity, the conversion of pressure and fugacity in this paper was carried out through the Peng–Robinson equation [31].

## 3. Results and Discussion

In order to determine the amount of CH_4_ injected into the model constructed in Section 2.1 and the Van der Waals energy change under different conditions, adsorption simulations of CH_4_ under 1–5 MPa at 283.15, 303.15, and 333.15K were carried out. Through simulation calculations, the adsorption characteristic parameters are shown in Table 1.

### 3.1. Adsorption Isotherm

**Figure 3:**
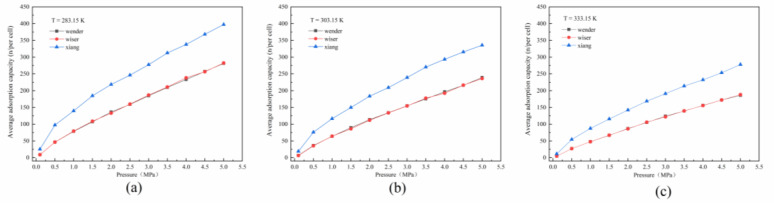
Isothermal adsorption curves of different coal samples at different temperatures: (**a**) 283.15 K, (**b**) 303.15 K, (**c**) 333.15 K.

The isothermal adsorption curves of the three coal samples with different metamorphic degrees under different temperature conditions are shown in Figure 3, and the corresponding average adsorption capacity is shown in Table 1. As shown in Figure 3, the adsorption capacities of methane increased with the increase in pressure under different temperature conditions, and the methane adsorption capacity corresponding to anthracite pores was the highest. At the same time, with the increase in temperature, the saturated adsorption amount of methane in coal pores with different metamorphic degrees showed a decreasing trend.

By comparing and analyzing the differences in methane adsorption capacity in Figure 3, we found that the isothermal adsorption curves of lignite and bituminous coal basically coincided at lower temperature conditions (281.15K, 303.15K), indicating that the adsorption capacities of lignite and bituminous coal (low-rank coal) were basically similar at the same pore scale, which agreed with the results of existing research [32,33]; the adsorption capacities of low-rank coal and medium-rank coal under the same environment conditions were similar. At the same time, the methane adsorption capacity of coal samples with a higher metamorphic degree and of the same pore size was significantly higher than that of coal samples with a lower metamorphic degree. By comparing the differences in methane adsorption capacity under different temperature conditions, we saw that the adsorption capacities of lignite and bituminous coal at 303.15 and 333.15K were similar, while the adsorption capacity of anthracite at 333.15K was significantly lower than that at 303.15K, indicating that under the same pore-size condition, the methane adsorption capacity of coal with a higher metamorphic degree was more significantly affected by temperature than that of lower-rank coal.

### 3.2. Energy Analysis

Adsorption energy can reflect the change in total energy before and after adsorption and characterize the stability of the adsorption system [19]. Many studies have shown that the adsorption of methane on the coal surface is mainly physical adsorption, primarily caused by the Van der Waals force between the methane molecules and coal molecules [34]. Based on these results, this paper analyzed the change trends of Van der Waals energy and electrostatic energy in different adsorption systems and further explored the change rule of interaction energy affecting adsorption. In this study, total energy was defined as the sum of intermolecular interaction energy and electrostatic energy. Since the influence of electrostatic energy on the total adsorption energy of the system was small, and the electrostatic energy was low, the electrostatic energy under different environmental conditions was less than −1.4 kJ/mol. Therefore, the change in electrostatic energy was no longer analyzed in this paper.

**Figure 4:**
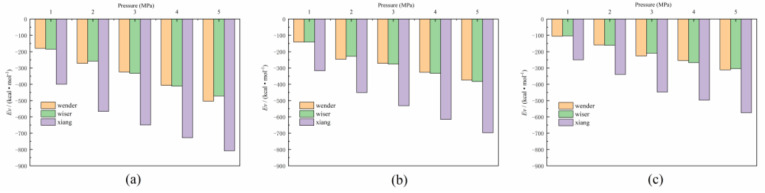
The Van der Waals energy of adsorption configurations: (**a**) 283.15 K, (**b**) 303.15 K, (**c**) 333.15 K.

Figure 4 shows the change in the Van der Waals energy in the adsorption systems under different temperature and pressure conditions. Obviously, the variation law of Van der Waals energy was basically consistent with the variation law of adsorption capacity shown in Figure 3. At a certain temperature, with the increasing pressure, the Van der Waals energy showed a downward trend. The anthracite with a high metamorphic degree under different conditions had the lowest Van der Waals energy; thus, the adsorption between CH_4_ and the coal matrix in high metamorphic coal samples was stronger, which was consistent with the results of most experiments on the difficulty of desorption of CH_4_ in high-rank coal. With the rise in temperature, the Van der Waals energy increased, and the interaction force weakened between different rank coals and methane molecules. Higher temperatures increased the kinetic energy of methane molecules, making adsorption more difficult and reducing the amount of adsorption. Therefore, it can be inferred that when the gas is extracted from coal seams, the relatively high temperature is beneficial to the desorption of CH_4_ to a certain extent. The results of this study were consistent with the results of Bai et al. [24].

### 3.3. Relative Concentration Distribution

**Figure 5:**
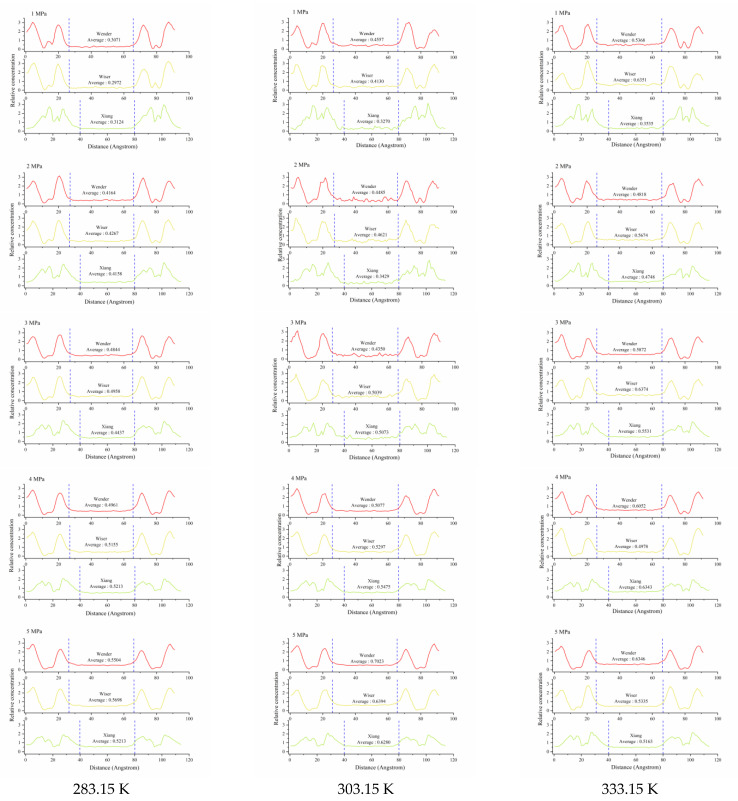
The relative concentration distributions of CH_4_ at different temperatures and pressures.

The methane adsorption configurations under different temperatures and pressures were analyzed to further clarify the distribution of pore methane in coal with different metamorphic degrees. As the established coal pore structure was symmetrical, in which the upper and lower regions belonged to the coal molecular layer and the middle region was the vacuum layer, the overall methane concentration distribution was symmetrical. The average concentration distributions of CH_4_ in the coal vacuum layer under different conditions are shown in Figure 5.

As shown in Figure 5, the blue dotted line area in the figure represents the 4 nm slit pore structure. Obviously, the relative concentration of CH_4_ in the vacuum layers increased with the increase in temperature, and the higher pressure corresponded to the higher relative concentration. Compared with the difference between different coal ranks, when the pressure was in the range of 1−5 MPa, the anthracite always with the lower relative concentration. However, as shown in Table 1 and Figure 3, the average adsorption capacity of anthracite was the highest in different environments; thus, it can be inferred that anthracite has a largely carbon-dominated surface, and there were more micropores in the coal matrix [32], resulting in the increase in adsorption capacity. At the same time, the three coal samples formed wave peaks near the slit, indicating that the slit wall had a strong adsorption effect on methane molecules. There were two to three wave peaks in the concentration distribution diagram in Figure 5, indicating that there was multilayer adsorption around the pore wall.

### 3.4. Diffusion Laws of CH_4_ in Coal

In order to characterize the migration characteristics of methane in pores of coal with different metamorphic degrees, this paper used the Forcite module in Material Studio 8.0 to calculate the dynamics of the system and then used the analysis task to calculate the mean square displacement (MSD) of methane to characterize the movement law of gas molecules under different conditions. The description of mean square displacement is shown in Formula (4) [35].

(4) $$r^{2}(t)=\frac{1}{N}\left[\sum_{i=1}^{N}{\left|r_{i}(t)-r_{i}(0)\right|}^{2}\right]$$

where $r_{i}\left(t\right)$ represents the position vector of particle i at the initial time (dimensionless), $r_{i}\left(0\right)$ represents the displacement of particle i at t = 0 (dimensionless), and *N* represents the number of adsorbed molecules.

The diffusion coefficient D is calculated based on the calculation results of mean square displacement combined with the Einstein algorithm, as shown in Formula (5) [35].

(5) $$D=\frac{1}{6N}\lim\frac{d}{dt}{\left\{\sum_{i=1}^{N}\left[r(t)-r(0)\right]\right\}}^{2}$$

The slope k is obtained by linear regression of the mean square displacement curve, as shown in Formula (6), and the diffusion coefficient can be simplified to Formula (7):

(6) $$k=\underset{t\to \infty }{\lim}\frac{1}{t}\left\{\frac{1}{N_{t}}\sum_{i=1}^{N}{\left|r_{i}(t)-r_{i}(0)\right|}^{2}\right\}$$

(7) $$D=\frac{k}{6}$$

**Figure 6:**
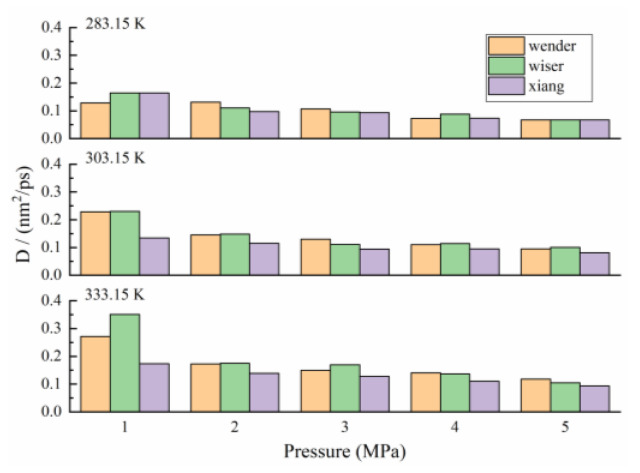
CH_4_ diffusion coefficients at different temperatures and pressures.

The influence of coal metamorphic degrees on the methane diffusion coefficient was comprehensively compared. As shown in Table 2 and Figure 6, with the increase in temperature, the diffusion coefficient of CH_4_ in coal pores increased, and it decreased with the increase in pressure, which was consistent with the existing research results [24]. Comparing the diffusion coefficients of CH_4_ in different metamorphic degrees of coal pore, the Xiang model (anthracite) under different conditions was the lowest. When the temperature was 283.15 K, the difference of the diffusion coefficients among different coal samples were small, which is about 0.067 nm^2^/ps at 5 MPa. However, the difference was gradually obvious with the increase in temperature. When the temperature was 333.15 K, the diffusion coefficient of high-rank coal (Xiang model) was only 0.0933 nm^2^/ps at 5 MPa. The corresponding diffusion coefficient of low-rank coal (Wender and Wiser models) was 0.1178 nm^2^/ps, and the reason for this phenomenon was related to the structural characteristics of coal. In the pore structure of the same scale, the diffusion performance of CH_4_ in the high-rank coal was obviously lower than that in the low-rank coal. It can be inferred that the molecular structure of the coal pore of the high-rank coal was easier to adsorb CH_4_ than that of the low-rank coal, and it was not easy to desorb after adsorption saturation, which corresponded to the strong Van der Waals interaction between the high-order coal and CH_4_. Therefore, it was necessary to adopt the interference of external environmental factors according to the structural characteristics of coal to achieve high-efficiency gas extraction.

## 4. Conclusions

Under the same pore-size condition, the higher-rank coal has a better adsorption capacity. With the increase in pressure, the interaction force between CH_4_ and different metamorphic grade coal increased, and the adsorption capacity increased. The high temperature increased the kinetic energy of the methane molecules, resulting in less adsorption capacity.

The diffusion coefficient of CH_4_ in high-rank coal was obviously lower than that of low-rank coal, which is corresponded to the strong Van der Waals force between CH_4_ and coal. At the same time, the methane adsorption capacity of high-rank coal was higher, and it was not easy to desorb after adsorption saturation, indicating that other interference methods were needed to realize subsequent efficient gas extraction.

The research results will provide theoretical support for the optimization of the gas extraction parameters of coal with different metamorphic degrees. However, we selected only three typical coal sample models and one pore size scale, which does not have wide applicability. Therefore, different coal structural models should be constructed and optimized according to the actual situation of the mine so as to optimize the gas extraction parameters.

## Figures and Tables

**Table 1:** Characteristic adsorption parameters of coal models containing adsorbed methane.

Coal Samples	T ^1^	P ^2^	Q ^3^	E_vdW_^4^	T	Q	E_vdW_	T	Q	E_vdW_
Wender	283.15	1	78.4002	−179.5581	303.15	63.6366	−141.04313	333.15	47.7494	−105.4962
Wiser			79.4713	−185.0184		63.9987	−141.0229		47.8132	−102.8283
Xiang			139.5387	−399.5742		116.6083	−317.6438		87.4664	−249.4977
Wender		2	136.1117	−271.6324		113.5411	−246.6959		87.0326	−158.7563
Wiser			132.9195	−258.2213		111.8657	−228.2721		85.9271	−160.3511
Xiang			218.7014	−566.3772		183.4784	−451.2131		142.2486	−339.69111
Wender		3	184.5834	−325.0988		154.8854	−271.6868		124.0636	−226.7707
Wiser			186.8574	−332.8752		154.6254	−275.7122		121.8778	−209.4812
Xiang			277.7498	−649.6994		239.2534	−531.8532		191.3180	−447.8494
Wender		4	233.1210	−407.0464		196.5561	−326.6914		156.0592	−254.8214
Wiser			238.1569	−411.5075		192.4109	−332.7546		155.7692	−266.9878
Xiang			337.8066	−727.7002		293.3652	−615.4051		232.2581	−496.8911
Wender		5	281.5214	−503.7776		239.0463	−374.3551		186.2385	−311.3128
Wiser			282.7054	−471.6480		236.0646	−383.1802		188.2769	−304.0934
Xiang			397.5014	−807.9002		335.8903	−696.9941		277.8015	−574.7722

^1^ T—Temperature, K. ^2^ P— Pressure, MPa. ^3^ Q—Average adsorption capacity, n/per cell. ^4^ E_vdW_—Van der Waals interaction energy, kcal/mol.

**Table 2:** CH_4_ diffusion coefficients of coal models containing adsorbed methane.

Coal Samples	T ^1^	P ^2^	D ^3^	T	D	T	D
Wender	283.15	1	0.1285	303.15	0.2282	333.15	0.2713
Wiser			0.1642		0.2301		0.3511
Xiang			0.1598		0.1341		0.1736
Wender		2	0.1311		0.1454		0.1726
Wiser			0.1103		0.1484		0.1748
Xiang			0.0975		0.1153		0.1387
Wender		3	0.1065		0.1299		0.1494
Wiser			0.0962		0.1112		0.1698
Xiang			0.0939		0.0943		0.1282
Wender		4	0.0733		0.1102		0.1404
Wiser			0.0884		0.1147		0.1365
Xiang			0.0731		0.0949		0.1098
Wender		5	0.0675		0.0948		0.1178
Wiser			0.0672		0.1004		0.1049
Xiang			0.0673		0.0811		0.0933

^1^ T—Temperature, K. ^2^ P—Pressure, MPa. ^3^ D—Diffusion coefficient, nm^2^/ps.

## Data Availability

Not applicable.
